# Supplementary figures and images for: EAGLE: An algorithm that utilizes a small number of genomic features to predict tissue/cell type-specific enhancer-gene interactions
Source: PLoS Comput Biol. 2019 Oct 30;15(10):e1007436. doi: 10.1371/journal.pcbi.1007436 (PMC6821050; doi:10.1371/journal.pcbi.1007436)

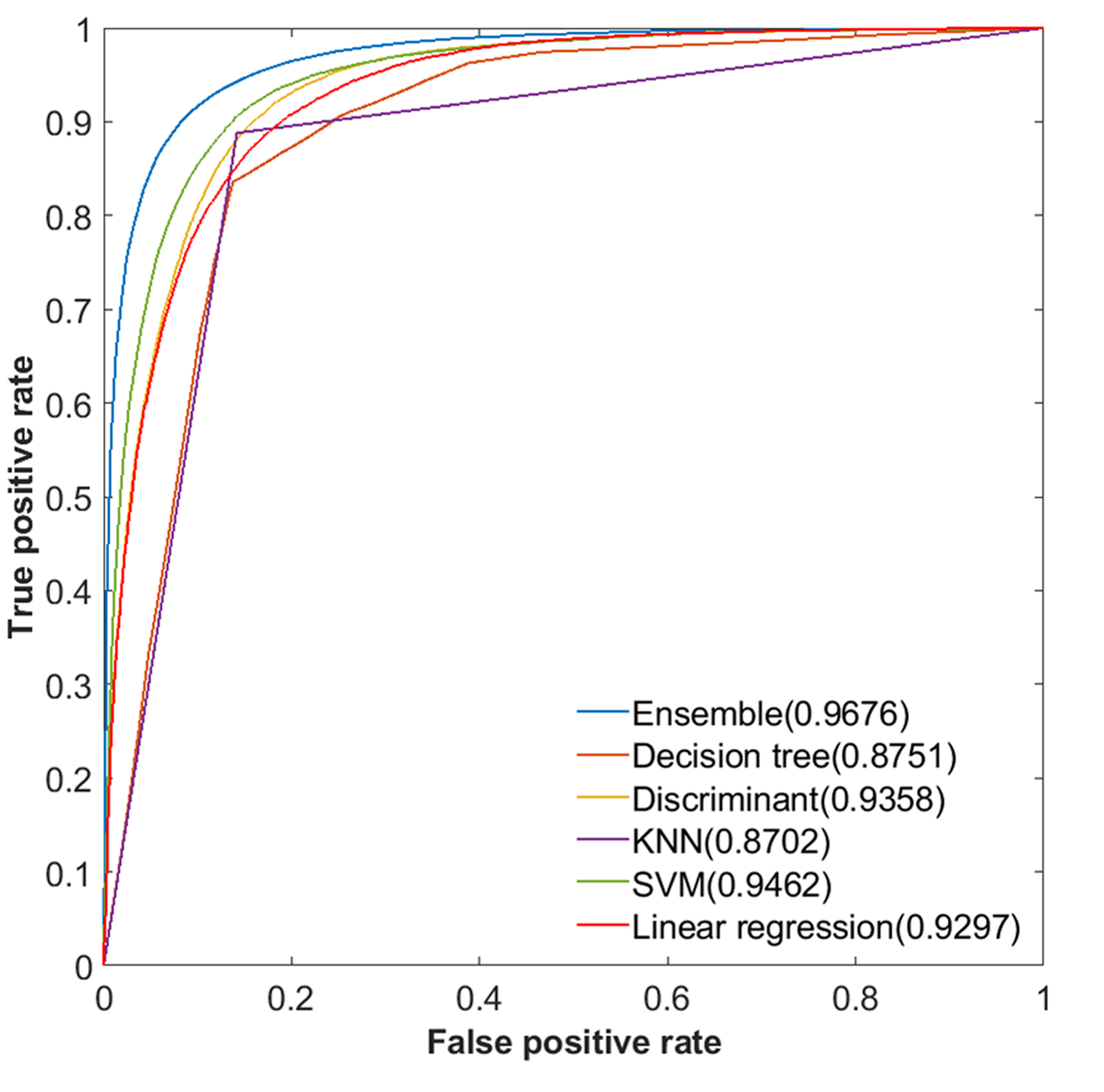

Supplement: S1 Fig — 71118 pairs with 35559 positives and 35559 negatives in K562 were taken as the common training data. 10-fold cross validation was used for all approaches. The functions “fitcensemble”, “fitctree”, “fitcdiscr”, “fitcknn” and “fitcsvm” in matlab, and “lm” in R were adopted to EAGLE, Decision tree, Discriminant, KNN, SVM, and linear regression respectively. (TIF) [file pcbi.1007436.s001.tif]

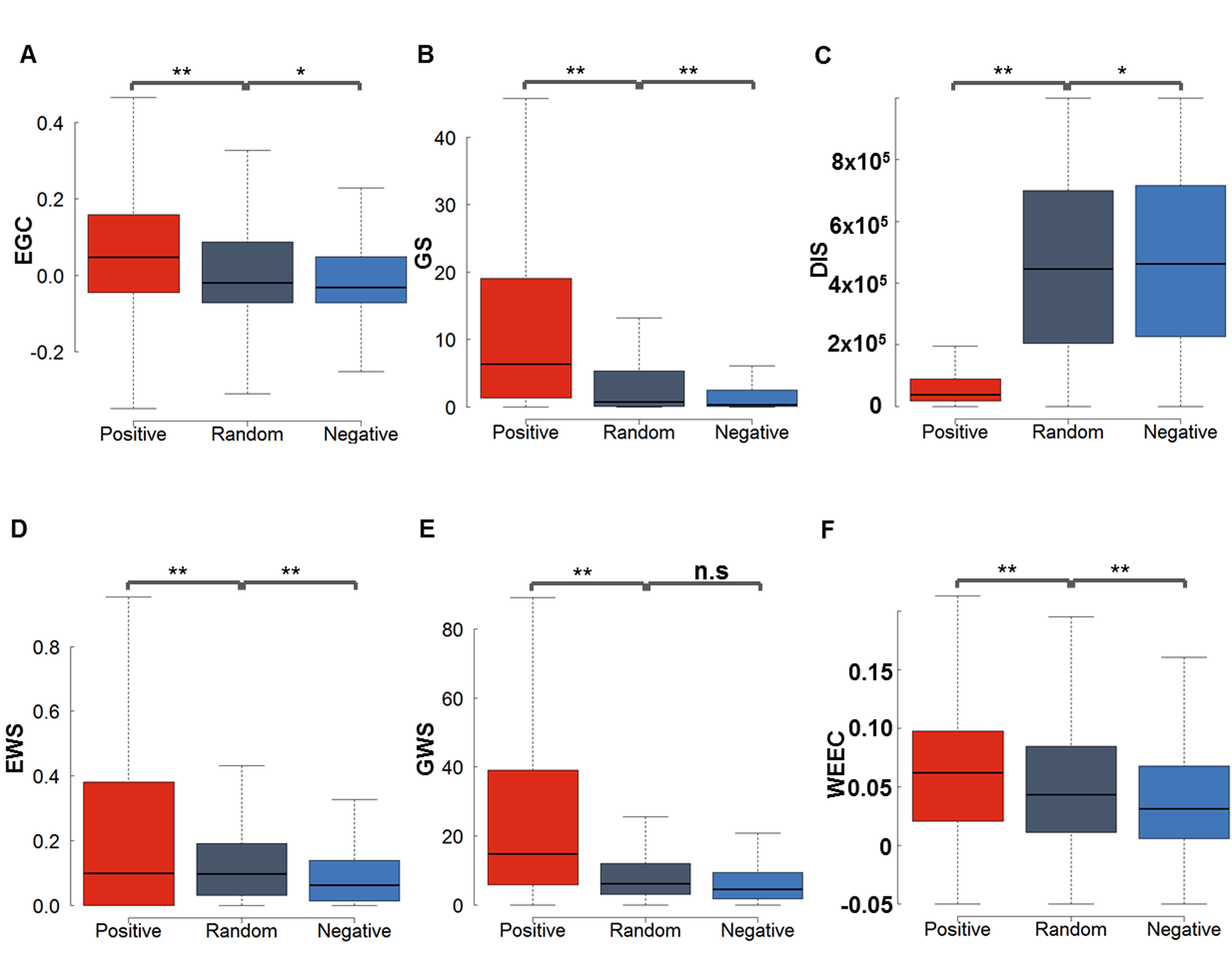

Supplement: S2 Fig — (A) Enhancer activity and gene expression profile correlation (EGC). (B) Gene score (GS) from the RNA-seq data. (C) Distance (DIS) between enhancer and gene in a pair. (D) Enhancer window signal (EWS) measuring the mean enhancer signal in the region between enhancer and promoter (E) Gene window signal (GWS) evaluating the mean gene expression level in the region between enhancer and promoter (F) The weight of enhancer-enhancer correlation (WEEC). The positive, negative and random enhancer-gene pairs were obtained from ChIA-PET dataset in K562. The P values were calculated using Student t test. (TIF) [file pcbi.1007436.s002.tif]

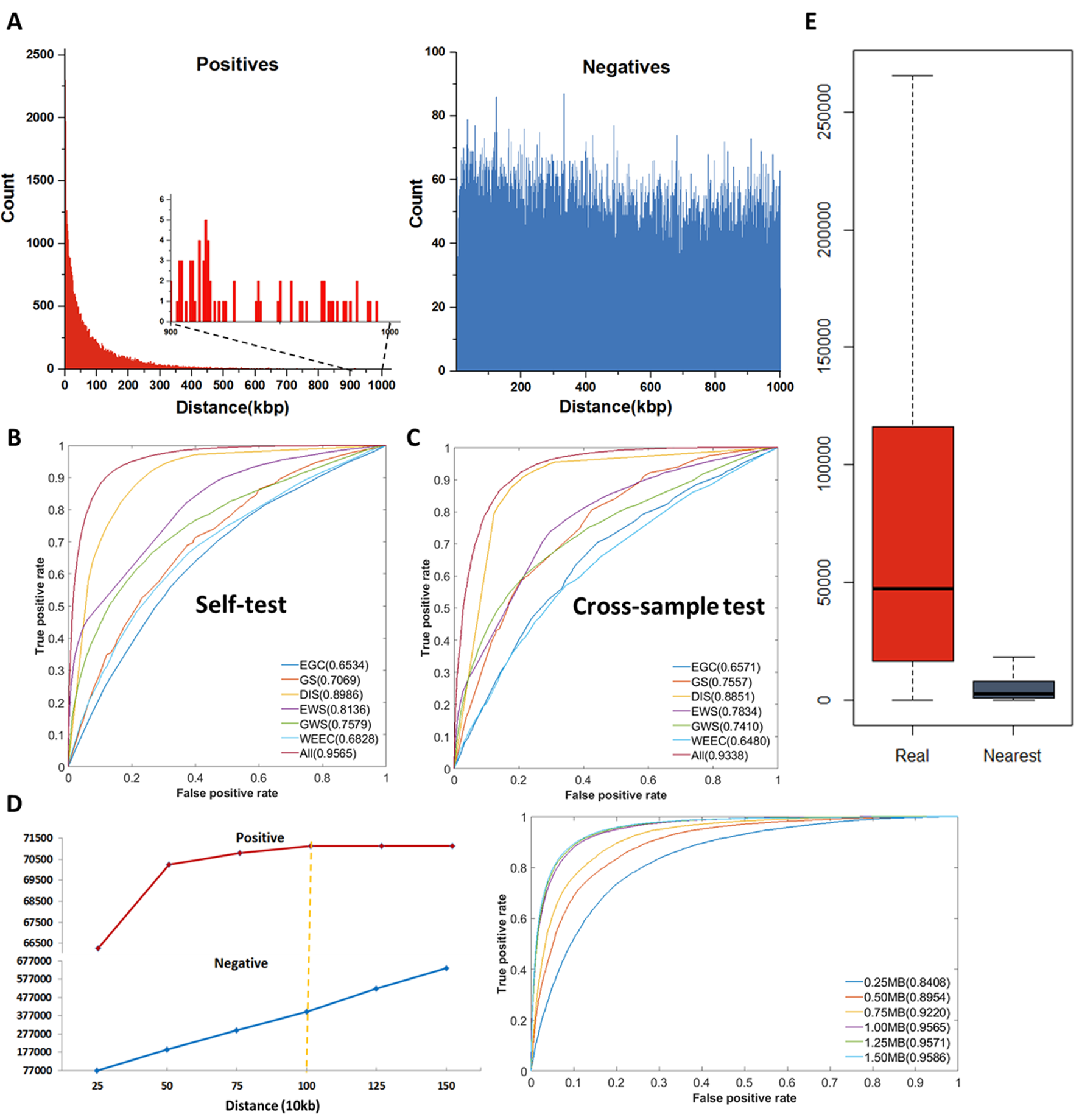

Supplement: S3 Fig — (A) Distributions of distances in positives and negatives of K562. (B) Individual self-test performance of DIS and other features in K562. (C) Individual cross-sample test performance of DIS and other features with training in K562 and testing in GM12878. (D) Changes of the number of positives/negatives and the prediction performances with various cutoffs of scanned regions. (E) Comparison of distances between positives (Marked as “Real”) and pairs with nearest genes in K562. (TIF) [file pcbi.1007436.s003.tif]

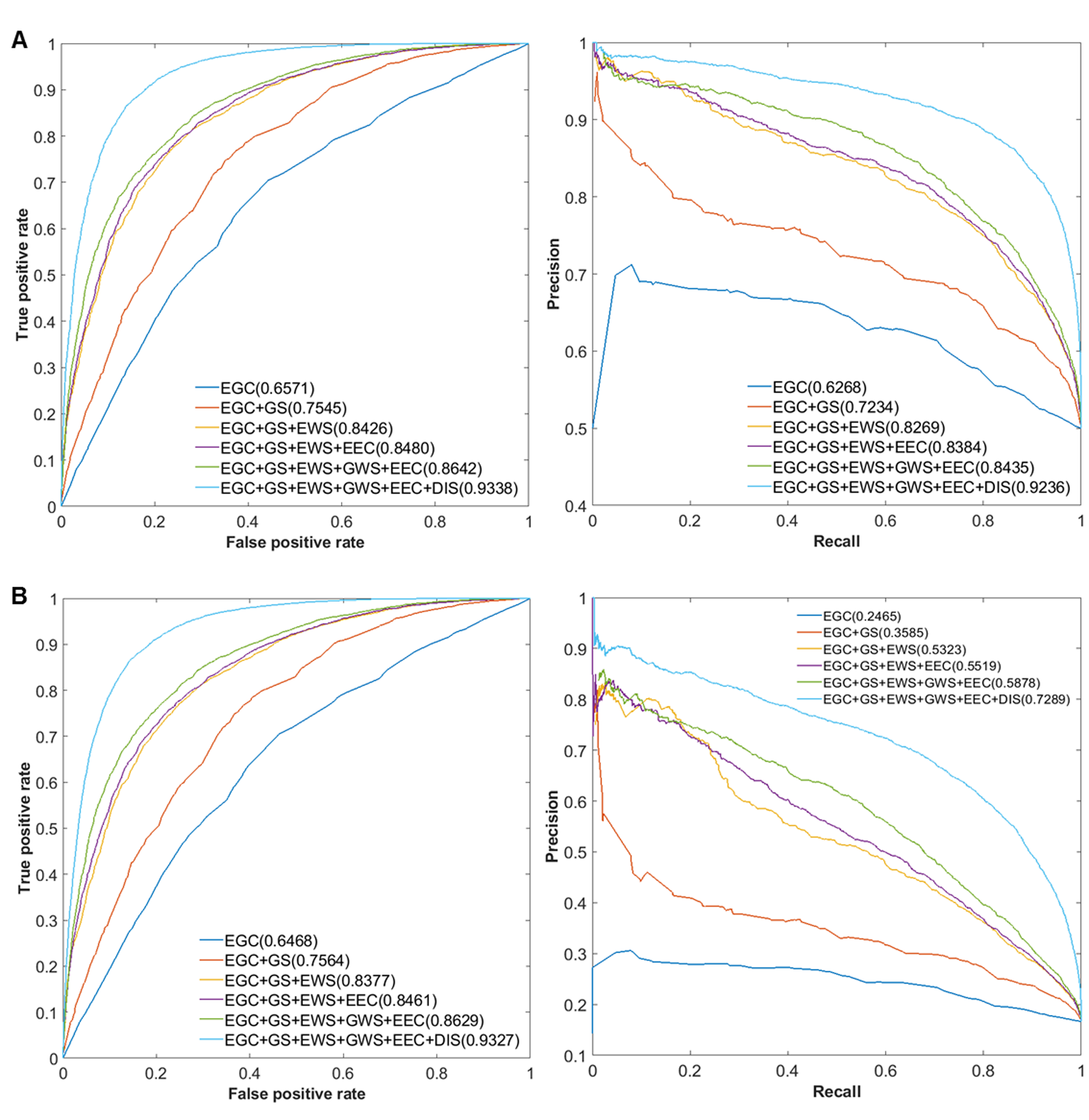

Supplement: S4 Fig — We trained the model using K562 and tested the model in GM12878. (A) Testing based on balanced data with 9732 positives and 9732 negatives in GM12878. Left panel is the ROC and right panel is the PR curves. (B) Testing using unbalanced data with 9732 positives and 48661 negatives in GM12878. Left panel is the ROC and right panel is the PR curves. We successively added the features (EGC, GS, EWS, GWS, EEC and DIS) to show the improving performance. (TIF) [file pcbi.1007436.s004.tif]

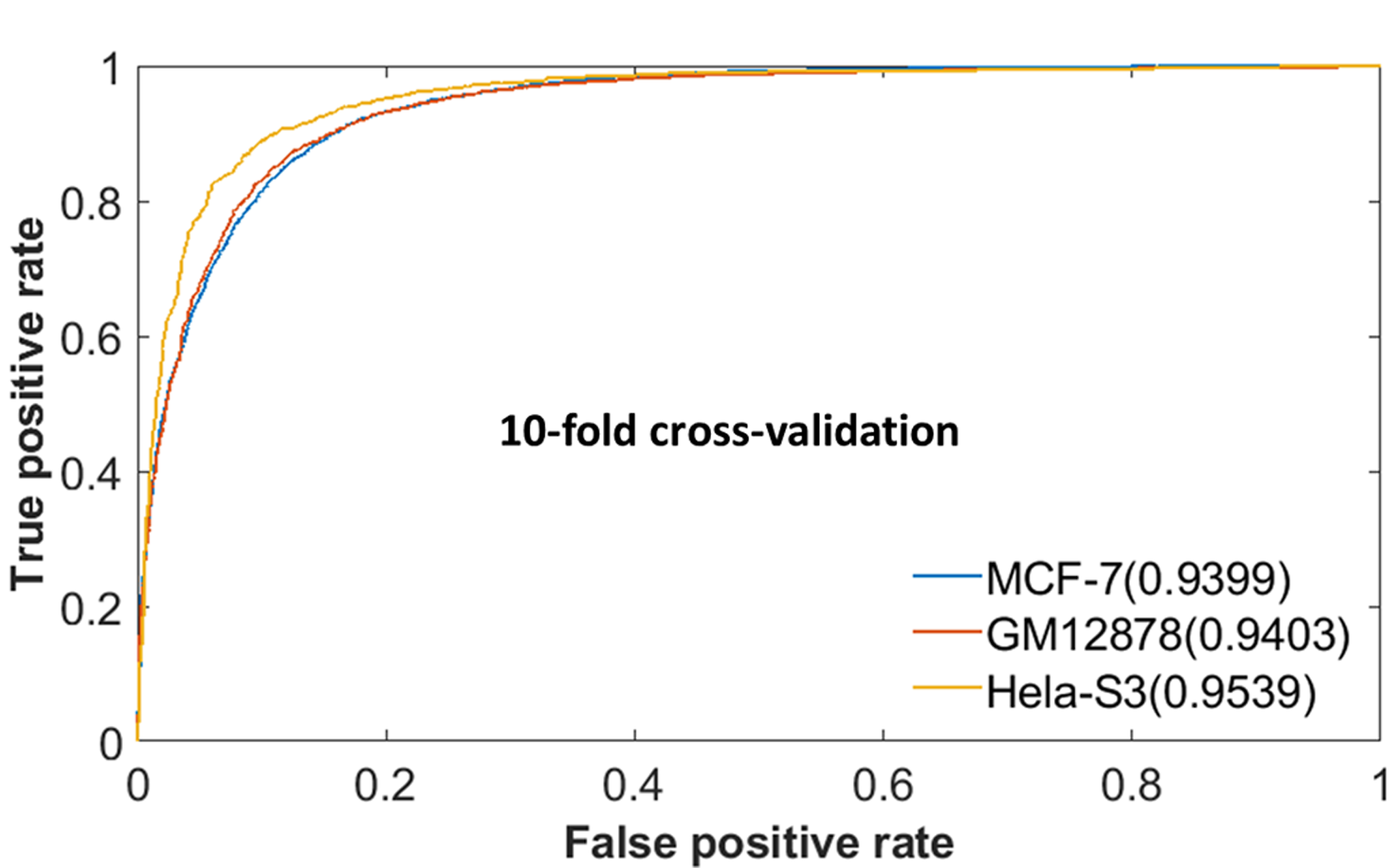

Supplement: S5 Fig — (TIF) [file pcbi.1007436.s005.tif]

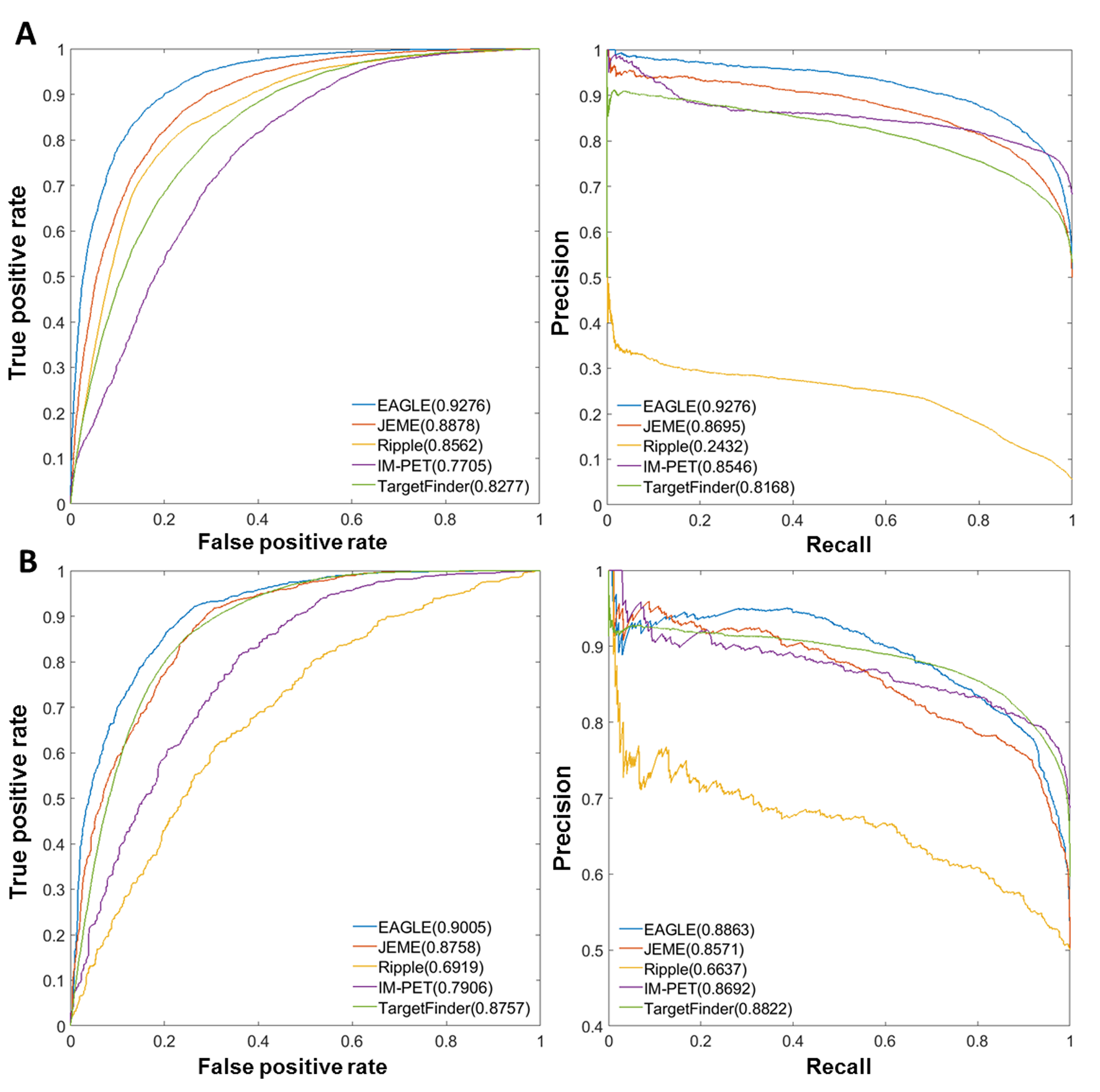

Supplement: S6 Fig — (A) Relative AUROCs and AUPRs of all tools in MCF-7 (B) AUROCs and AUPRs of five tools in Hela-S3. The cross-sample validation was performed with the training in K562 and testing in other cell lines (see Methods). (TIF) [file pcbi.1007436.s006.tif]

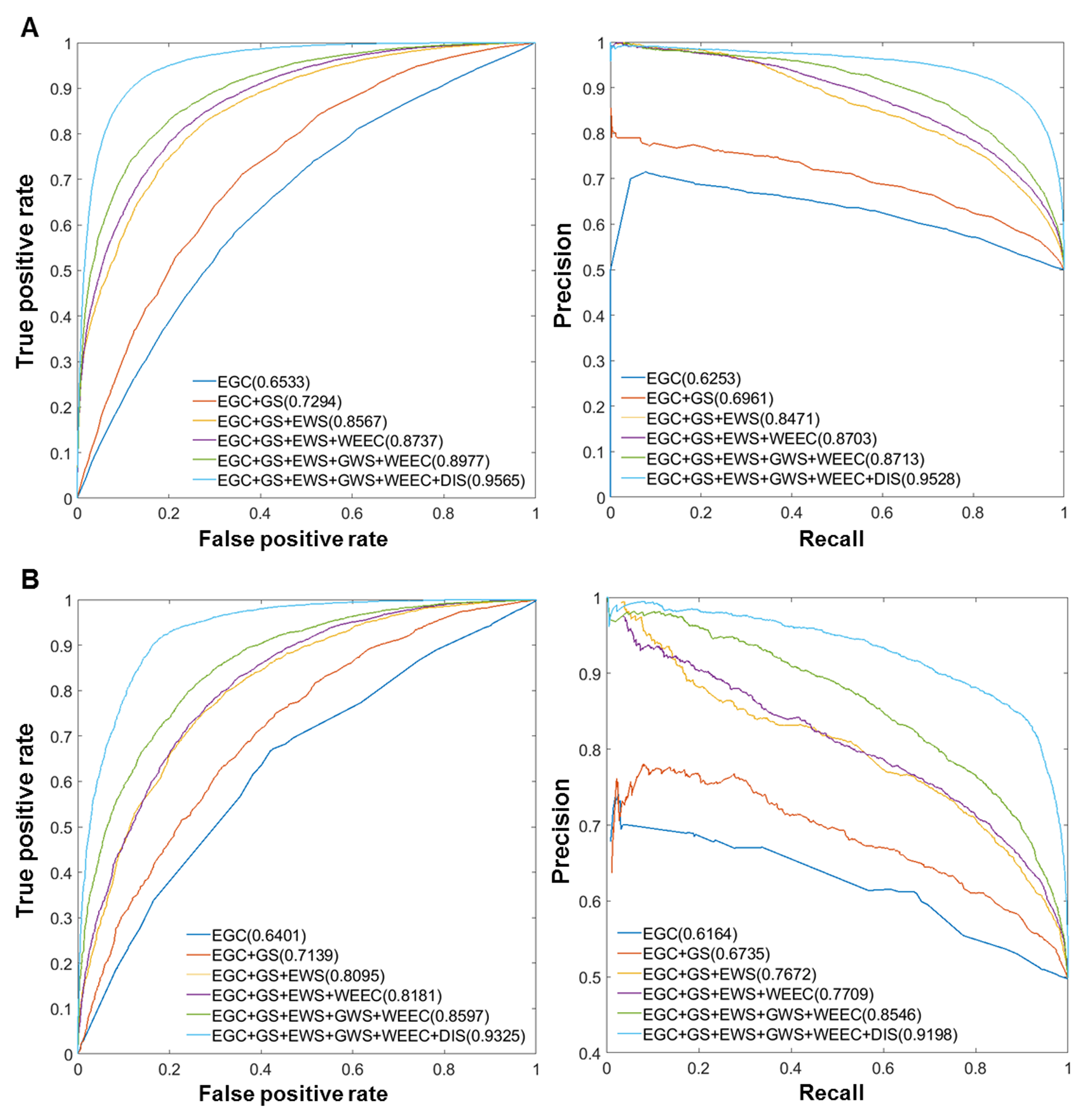

Supplement: S7 Fig — (A) Performances (AUROC and AUPR) gradually improved with successive adding of the training features in K562. (B) Performance (AUROC and AUPR) increasing with adding the training features one by one in MCF-7. For each cell line, the self-testing used one half of the data for training and the other half for testing. (TIF) [file pcbi.1007436.s007.tif]

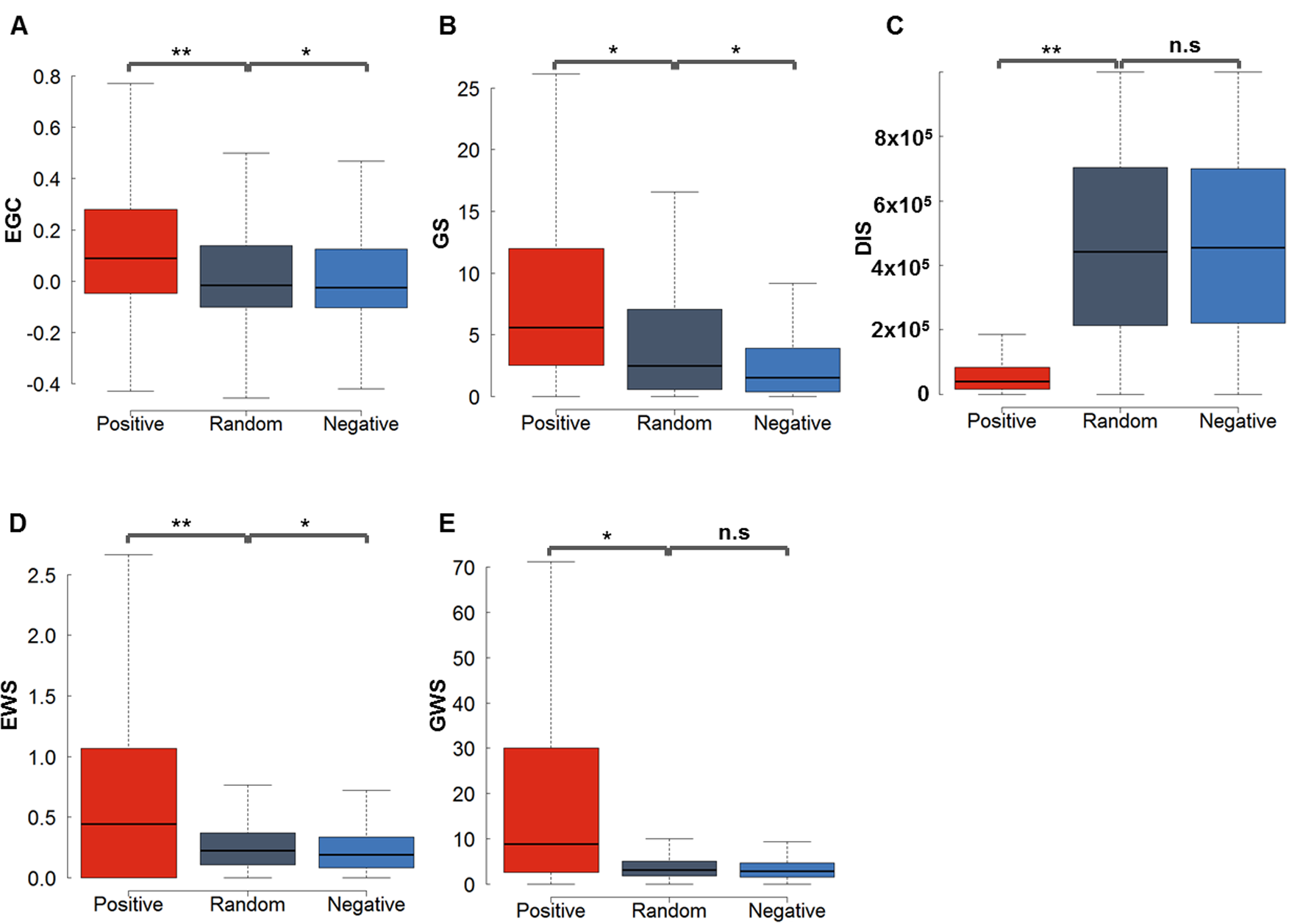

Supplement: S8 Fig — (A) Enhancer activity and gene expression profile correlation (EGC) (B) Gene signal from the RNA-seq data. (C) Distance between enhancer and gene in a pair. (D) Enhancer window signal measuring the mean enhancer signal in the region between enhancer and promoter (E) Gene window signal evaluating the mean gene expression level in the region between enhancer and promoter. The P values were calculated by the Student t test. (TIF) [file pcbi.1007436.s008.tif]

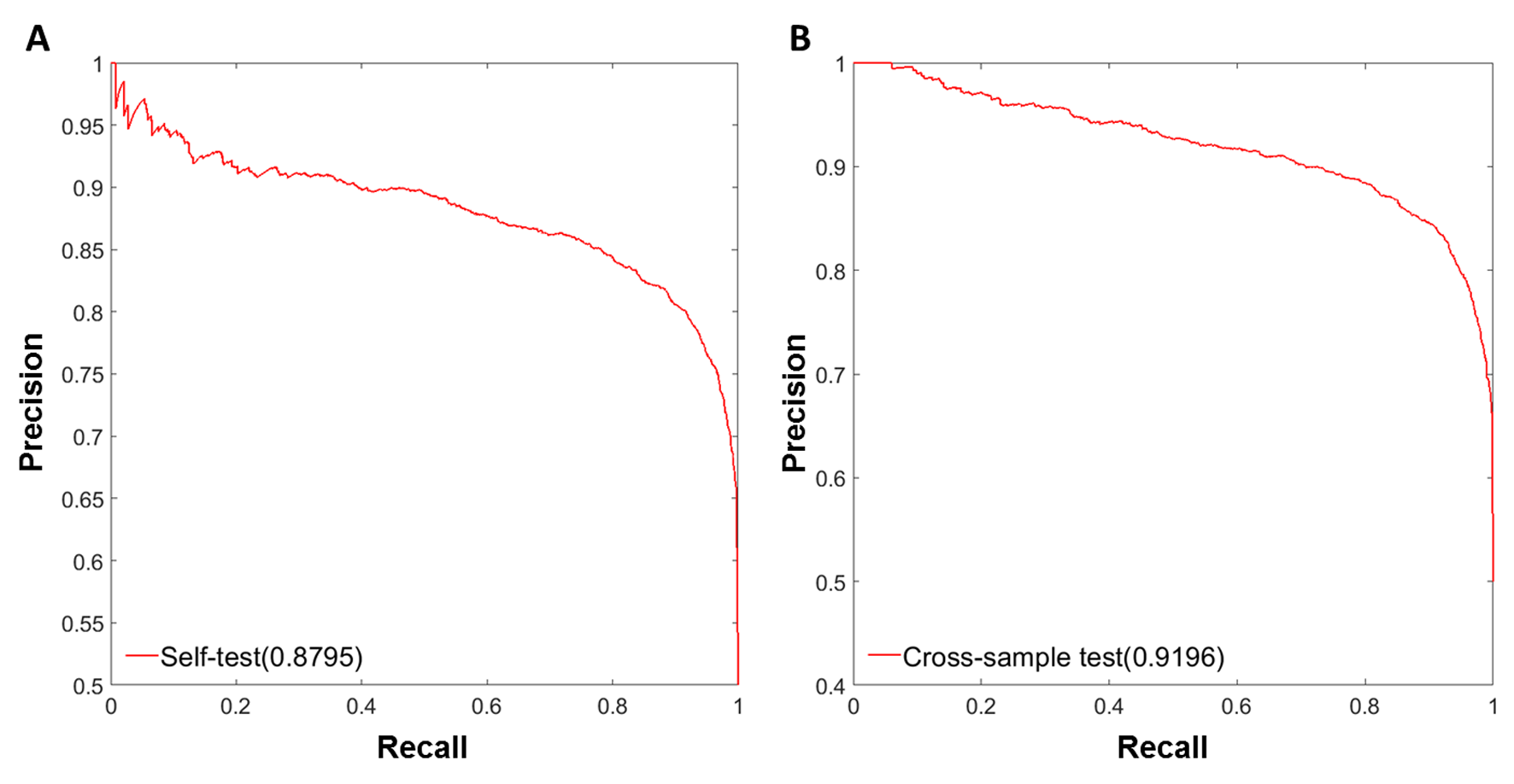

Supplement: S9 Fig — (A) Self-testing by PR plot in lung. (B) cross-sample test on spleen with PR plot by lung model. (TIF) [file pcbi.1007436.s009.tif]

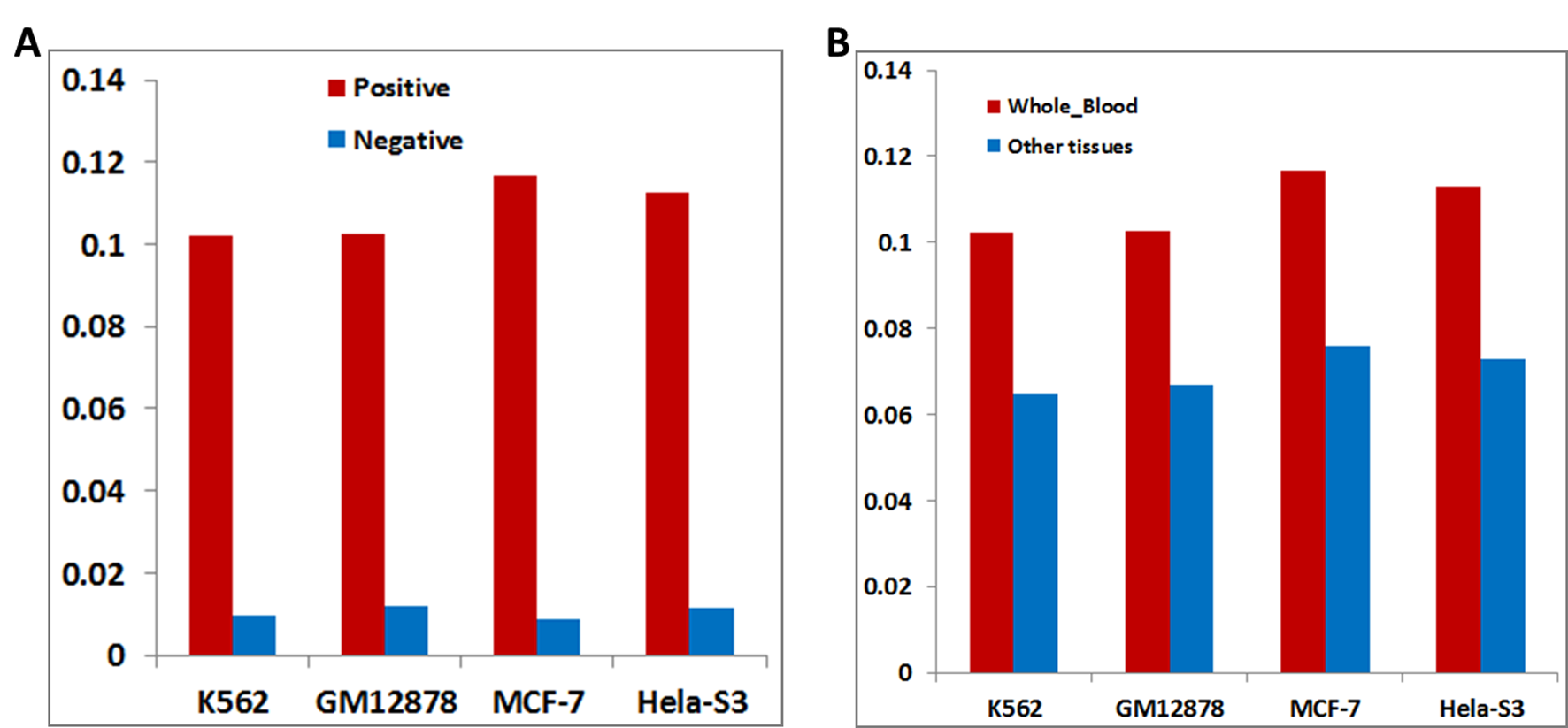

Supplement: S10 Fig — The enhancers and expression data in GM12878 were taken as the input. (A) The similar percent (around 11%) of positives and percent (around 0.7%) of negatives in the predicted EG interactions of GM12878 by different models, overlapping with eQTLs in whole blood. (B) The simimar percent (around 11%) of positives overlapping with whole blood eQTLs much higher than that (~7%) in other 47 tissues. (TIF) [file pcbi.1007436.s010.tif]

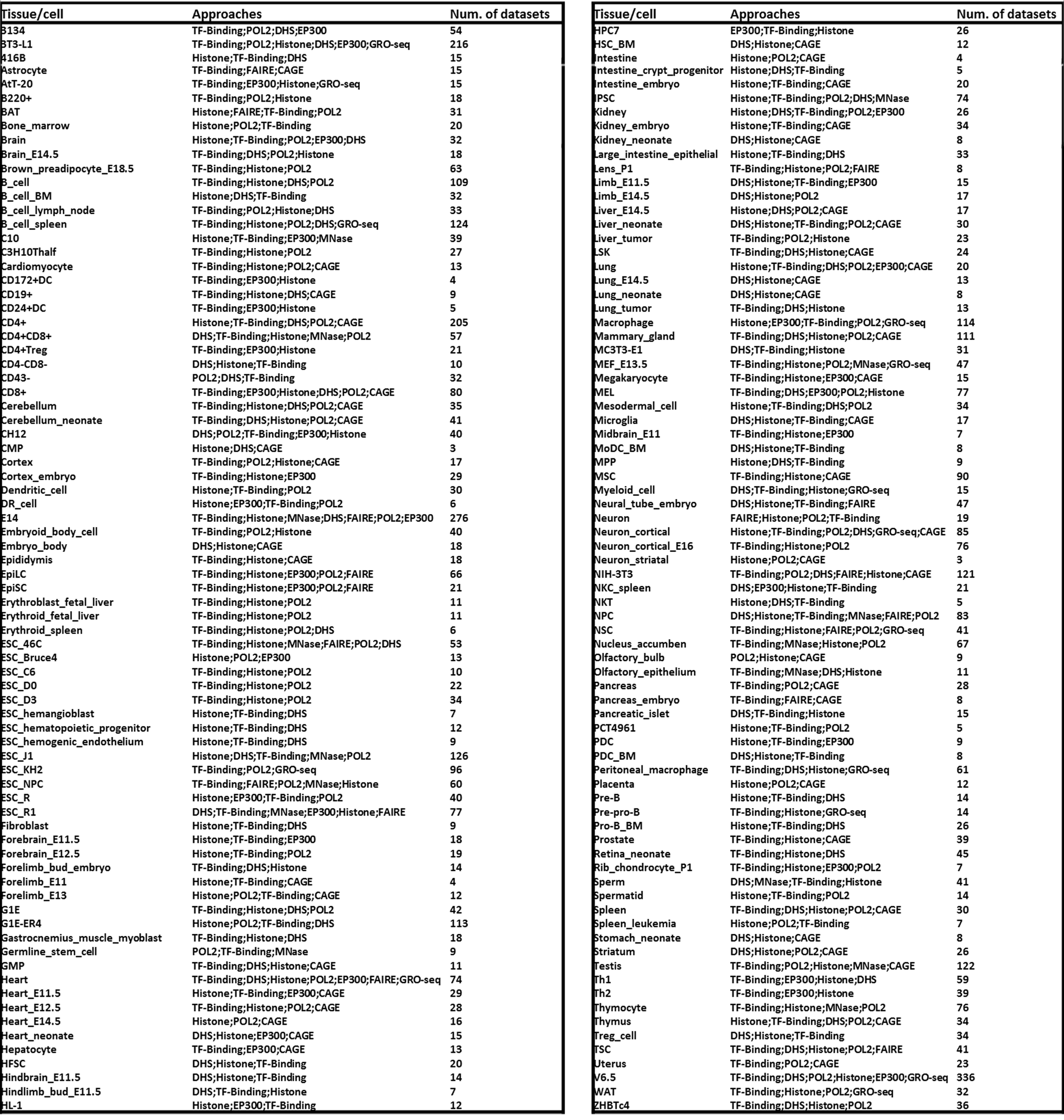

Supplement: S1 Table — Each tissue/cell type has at least three tracks and each enhancer is supported by at least one half of the tracks in the relative tissue/cell type. (TIF) [file pcbi.1007436.s012.tif]
